# Effect of eight weeks of interval training on insulin signaling and neurodegeneration in the hippocampus of Methamphetamine-treated rats

**DOI:** 10.1038/s41598-026-41118-w

**Published:** 2026-02-27

**Authors:** Ahad Shafiei, Amir Hossein Haghighi, Majid Asadi-Shekaari, Mahla Sadat Nabavi-Zadeh, Roya Askari, Hamid Marefati, Hadi Shahrabadi, Alfredo Caturano, Rosario Barone, Roberto Bei, Pasquale Farsetti, Attilio Parisi, Ferdinando Iellamo, Marco Alfonso Perrone

**Affiliations:** 1 Department of Physical Education and Sport Sciences, Kerman Branch, Islamic Azad University, Kerman, Iran; 2 Department of Exercise Physiology, Faculty of Sport Sciences, Hakim Sabzevari University, Sabzevar, Iran; 3 Neuroscience Research Center, Neuropharmacology Institute, Kerman University of Medical Sciences, Kerman, Iran; 4 General Department of Sports and Youth of Kerman Province, Kerman, Iran; 5 Department of Human Sciences and Promotion of the Quality of Life, San Raffaele Roma Open University, Rome, 00166 Italy; 6 Department of Biomedicine, Neurosciences and Advanced Diagnostics, University of Palermo, Palermo, 90127 Italy; 7 Department of Clinical Sciences and Translational Medicine, University of Rome Tor Vergata, Rome, 00133 Italy; 8 Department of Movement, Human and Health Sciences, University of Rome Foro Italico, Rome, 00135 Italy

**Keywords:** Methamphetamine, Neurodegeneration, Aerobic training, APP, Tau, Diseases, Neurology, Neuroscience, Physiology

## Abstract

This study investigated the effect of eight weeks of interval training on insulin signaling and neurodegeneration in the hippocampus of methamphetamine (METH)-treated rats. Thirty-two male Wistar rats were randomly divided into four equal groups (*n* = 8): saline, METH-1 (21-days of injection period), METH-2 (21-day injection period, followed by 8-week withdrawal), and METH+moderate-intensity interval training (MIT). Methamphetamine was injected intraperitoneally at 5 mg/kg per day for a period of 21 consecutive days. MIT was conducted on a treadmill at 60–65% of maximum speed, 5 days weekly for 8 weeks. At the end of the injection and training period, the hippocampal tissue of rats was extracted to evaluate the pathological changes and gene expression of related indicators. METH injection caused a disturbance in the insulin signaling pathway, resulting in increased neurodegeneration, as evidenced by a decrease in the expression of IRS-1 (*p* < 0.001) and Akt (*p* < 0.001) genes as well as an increase in the expression of GSK-3β (*p* < 0.001), APP (*p* = 0.007), tau (*p* = 0.006), p-tau (*p* < 0.001), and Caspases-3 (*p* = 0.003) genes. However, MIT significantly upregulated the expression of IRS-1 (*p* < 0.001) and Akt (*p* = 0.002), while downregulating APP (*p* = 0.028) and p-tau (*p* < 0.001). These findings suggest that MIT enhances insulin signaling activity and improves anxiety-like behaviors, thereby counteracting the detrimental effects of METH. Further studies are required to expand and validate these findings.

## Introduction

Methamphetamine (METH) is an addictive stimulant frequently consumed by youth and adolescents^1^. The drug’s abuse potential is linked to its powerful stimulation of dopaminergic pathways in the central nervous system (CNS). Although the mechanisms of nerve cell degeneration due to METH consumption in the CNS are not exactly understood, different studies have shown that oxidative stress, induction of apoptosis, activation of microglia, hyperthermia, and other factors are involved^2^. Recent studies have investigated the role of METH and its effect on reducing the activity of the insulin signaling pathway in the CNS, thereby reducing the cognitive function and memory^3^.

Insulin receptors (IRs) are highly distributed in the olfactory bulb, hippocampus, and hypothalamus regions and initiate auto-phosphorylation of downstream activities, i.e., insulin receptor substrate (IRS) protein, which subsequently activates inositol triphosphate-kinase (PI3K) and protein kinase B (Akt). PI3K/Akt pathway in the brain is an important insulin signaling pathway for neuronal protection, learning, and memory^4^. Akt inactivates one of the key molecules downstream of the PI3K/Akt signaling pathway and plays a role in serious pathological conditions, including cognitive impairment related to Alzheimer’s disease (AD)^5^. A major hypothesis posits that defective processing of amyloid precursor protein (APP) initiates amyloid-beta (Aβ) plaque formation, which in turn activates downstream mechanisms leading to tau-pathology and thereby contributes to the progression of AD^6^. A recent study reported that repeated injection of mice with METH led to obvious cognitive defects in the Y-shaped maze behavioral test, along with insulin signaling disorder (IR/IRS2/PI3K/Akt/glycogen synthase kinase-3β (GSK3β))^3^. It has been demonstrated that METH exposure impairs insulin signaling pathways, leading to brain insulin resistance. This dysfunction is characterized by downregulation of IRS-1, decreased p-Akt levels, and concomitant activation of GSK-3β. Given that GSK-3β is a critical kinase for tau phosphorylation, its dysregulation promotes aberrant tau modifications^7^.

Several preventive strategies have been proposed for the neurotoxic effects of METH, including regular physical activity. Exercise training can reduce the prevalence and severity of AD by modulating key signaling pathways such as PI3K/Akt. These pathways interact to inhibit Aβ plaque formation, reduce tau hyperphosphorylation, decrease apoptosis and neuroinflammation, improve autophagy, and ultimately enhance cognitive function in individuals with AD^8^. In Alzheimer’s model mice, treadmill exercise at 45–55% of maximal oxygen uptake (VO_₂max_) led to a marked reduction in tau mRNA expression and the levels of tau, Aβ, and p-tau proteins, accompanied by increased PI3K and p-Akt protein levels, as well as improved learning and memory performance^9^. In addition, aerobic exercise was shown to induce a significant decrease in acetylated tau protein levels and phosphorylated GSK3βY216, accompanied by a significant increase in IRS-1 protein levels, as well as improvements in memory, motor, and balance functions in rats subjected to middle cerebral artery occlusion^10^. However, the role of intermittent exercise in reducing the damage caused by METH on the insulin signaling pathway in the hippocampus is not yet clear. This study aimed to investigate whether interval aerobic training could counteract hippocampal insulin signaling dysfunction and neurodegeneration induced by chronic METH administration.

## Materials and methods

### Animals

**Fig. 1:**
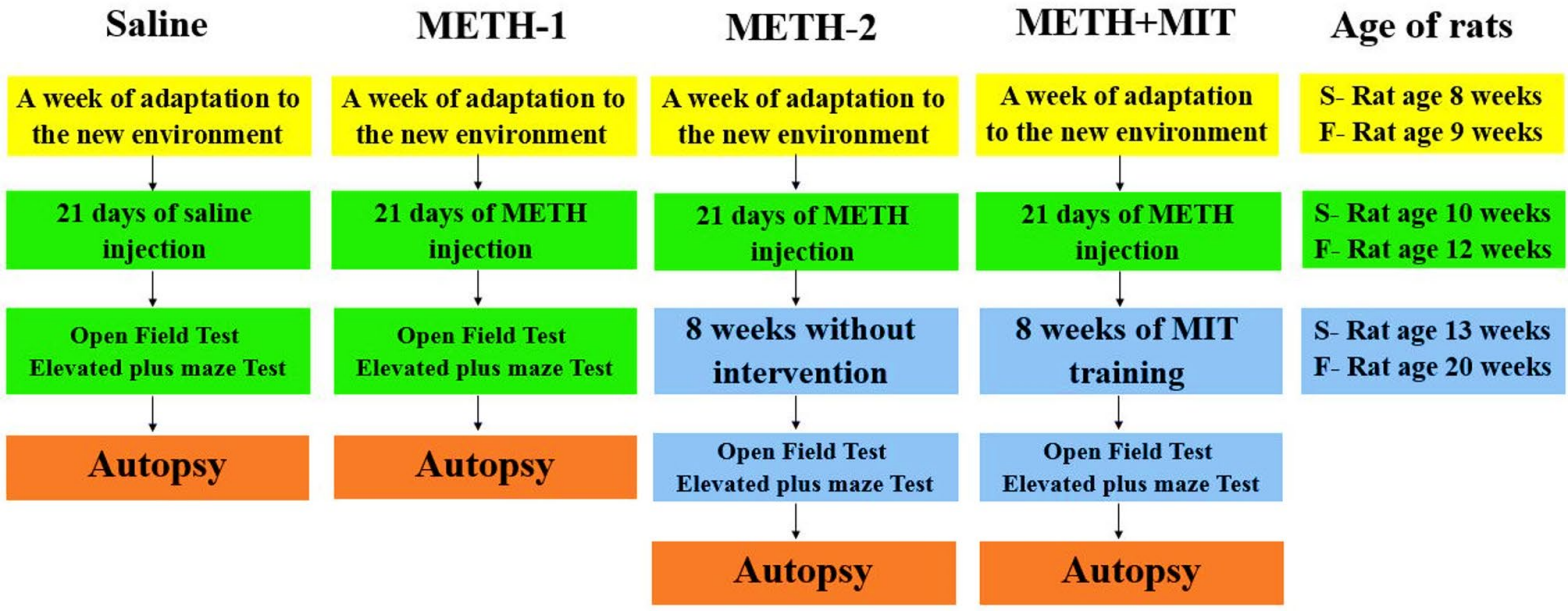
Schematic representation of the implementation of interventions in this study.

Male Wistar rats (8 weeks old, weighing 180–220 g) were purchased from Animal Farm of Kerman University of Medical Sciences, Iran and kept in a colony room at Kerman Neuroscience Research Center, Iran, with a 12:12 h light/dark cycle at 21 ± 2 °C and handled according to standard protocols for the use of laboratory animals. The rats were randomly divided into four equal groups (*n* = 8): saline, METH-1 (21-days of injection period), METH-2 (21-day injection period, followed by 8-week withdrawal), and METH+moderate-intensity interval training (MIT). The study complies with ARRIVE guidelines (PLoS Bio 8(6), e1000412,2010​). All procedures for animal treatment were approved by the Ethics Committee of Hakim Sabzevari University, Sabzevar, Iran (ethics code: IR.HSU.REC.1401.008). Figure 1 presents a schematic diagram summarizing the procedures used to carry out the interventions in this research.

### Drugs

METH hydrochloride (purity < 96% from Kerman National Drug Center, Iran) was dissolved in normal saline (0.9% injection). The METH groups received 5 mg/kg (i.p.) once daily for 21 days^11^. The administration of the drug was initiated at 10 weeks of age in the rats. The maximum total injection volume for the drugs was 1 ml.

## Exercise protocol

MIT (60–65% of maximum speed) was conducted on an enclosed rodent treadmill (Tread Mill 2021, PN, Tajhiz Gostare Omide Iranian) with an inclination of 0°. The protocol was applied 5 days per week for 8 weeks, commencing when the rats reached 13 weeks of age.

According to Table 1, the training duration in each session was 36 min, including 6 min of warm-up with an intensity of 20% of maximum speed, 6 min of cooling with an intensity of 20% of maximum speed, and the main program of MIT with an intensity of 60–65% of maximum speed. Before implementing the training protocol, an exhaustive sports test was performed, starting at a speed of 10 m/min, and for every 3 min, the speed was increased by 3 m/min^12^. Then, rats’ mean maximum speed in the METH + MIT group was calculated to perform the exercise program.

**Table 1:** MIT protocol in this research.

Weeks	1	2	3	4	5	6	7	8
Number of intervals	4	4	4	4	4	4	4	4
Effort duration (min)	4	4	4	4	4	4	4	4
Effort velocity (m/min)	16	18	20	22	24	26	28	30
Rest duration	2	2	2	2	2	2	2	2
Rest velocity (m/min)	10	10	11	11	12	12	13	13

MIT: moderate-intensity interval training.

### Open field test (OFT)

Open Field Test (OFT) assay evaluated the locomotion of the animals and anxiety-like behaviors. An opaque plexiglass box (90 cm × 90 cm × 45(H) cm) was used in the OFT procedure, and all animal’s behaviors were recorded using Ethovision Software (Noldus Technology, Netherlands). Animals were placed in the middle of the field, and the following parameters were recorded by the software for each rat during a five-minute interval: total distance moved (cm), speed, the number of rearing (animals standing upright on both hind legs in a vertical position), and grooming (rats licking reachable parts of their bodies). Increased distance traveled and time spent in the center of the arena are typically interpreted as reduced anxiety-like behavior and enhanced exploratory drive, whereas elevated grooming may reflect heightened stress or emotional arousal^13,14^. Behavioral assessments were performed over two days at the end of 12 and 20 weeks of age in rats.

## Elevated plus maze (EPM) behavioral test

To evaluate the level of anxiety, an Elevated Plus Maze (EPM) device was used as a standard model for evaluating the level of anxiety in rodents. EPM is a wooden device including two open arms (each 5 cm × 50 cm), two closed arms (each 5 cm × 50 cm × 40 cm (H)), and a central bottom (5 cm × 5 cm). The open arms face each other, and the closed arms face each other and are placed about 50 cm above the room floor. This experimental model of measuring anxiety is unconditional and does not require the training and learning of the animal. To measure the level of anxiety, the animal was placed in the EPM device (in the palm part and facing the open arm), and direct exploratory activities, the number of entries into the open arms, and the duration of time spent in the open arms were evaluated and recorded for five minutes during the day. An increase in entering the open arms and the time spent in them is considered an indicator of reducing anxiety in rats. A decrease or increase of both indicators (entering the open arms and the time spent in them) in the same direction, and at least a significant difference of one indicator with the control group, is considered as a significant change in the level of anxiety^15^.

## Pathological changes

Rat brains were carefully dissected from the skull without damaging the organ and were placed in 10% formaldehyde (MERCK, Germany) for fixation. After processing, tissue sections with a 5 microns thickness were prepared and stained with hematoxylin and eosin. Hippocampus photos were taken by an optical microscope (Olympus CX21FS1, Japan) equipped with a photography camera (Canon, Japan). The images were studied using an objective lens with a magnification of 40. The neuronal density (N/mm3) of the CA1 area of the hippocampus was calculated by the dissector method, which involved counting neurons in one reference four-stick. If a neuron was present in both four reference sticks, it was not counted in the count. However, if a neuron was present in four reference sticks, but not in the four sticks next to them, it was counted^16^.

### Tissue dissection and preparation for quantitative PCR analysis

For the molecular experiments, the animals were anesthetized with CO2 gas (100% CO2 in a 30 cm × 50 cm × 28 cm cylindrical Plexiglas chamber) and then sacrificed by decapitation. Both whole frontal cortices were rapidly dissected out on ice and frozen in liquid nitrogen. The dissected hippocampi from each rat were randomly distributed for further PCR assays and stored at − 80 °C until homogenization.

### Quantitative RT-PCR

The hippocampal tissue (50 mg) was lysed for RNA extraction using TRIzol solution (Yektatajhiz, Cat No: YT9066) (Unique Tajhiz Azma Company) and completely homogenized by a tissue homogenizer. RNase-DNase-free was used to remove DNA contaminants. All samples were measured using a Picodrop device (Picodrop Limited, Hinxton, UK) to measure RNA concentrations with wavelengths of 260/280 and 230/280.

cDNA synthesis was performed using the cDNA Synthesis Kit (Yektatajhiz, Cat No: YT4500) following the manufacturer’s protocol. The RNase inhibitor was added for decontamination, and cDNA synthesis was performed in a PCR device manufactured by Analitik Jena.

The expression level of the relevant genes was determined by real-time PCR (quantitative reverse transcription (qRT)-PCR) using commercial Real Q Plus 2 × Master Mix Green enzyme (Ampliqon SYBR green Master Mix High ROX, Cat No: A323402, Denmark) (Real-time PCR of Rotor-Gene Q model made by QIAGEN company). Initial denaturation was at 95 °C for 15 min, followed by 40 consecutive cycles of denaturation at 95 °C for 10 s, 60 °C for 20 s, and 72 °C for 20 s.

The primer sequences were designed by Primer-BLAST (NCBI) online software, and the gene Gapdh was used as an internal control gene (Table 2). Data analysis was performed based on threshold cycle comparison (CT). The amplification curve of each PCR reaction was normalized with the amplification curve of the corresponding Gapdh reference gene. The CT difference obtained from the tested and control samples was calculated, and the ratio of the target gene to the reference gene was calculated using the CT-2 formula.

**Table 2:** The specifically designed primers and sequences of the reference gene for the quantitative real-time PCR.

Target	Forward Primer	Reverse Primer	TM(c)
IRS-1	5’-CCTCACCAACCCTTAGGCAG-3’	5’-GTCTTTCATTCTGCCTGTGACG-3’	60.10
Akt	5’-CCCTTCCTTACAGCCCTCAAG-3’	5’-ACACAATCTCCGCACCGTAG-3’	60.2**5**
GSK-3β	5’- ACTCTACCTGAACAGCCCCA-3’	5’-AACGTGACCAGTGTTGCTGA-3’	58.60
APP	5’-GCGGCAACAGGAACAACTTT-3’	5’-TGCCGTCGTGGGAAACAC-3’	59.10
tau	5’-AAGAAGCAGGCATCGGAGAC-3’	5’-CCTTGGCTTTCTTCTCGTCA-3’	57.30
p-tau	5’-GACCAGGCCGGAGATTACAC-3’	5’-AGCTTGGTCCTCCATGTTCG-3’	58.62
Caspases-3	5’-GCAGCAGCCTCAAATTGTTGACTA-3’	5’-TGCTCCGGCTCAAACCATC-3	59.50
Gapdh	5’-CAACTCCTCAAGATTGTCAGCAA-3’	5’-GGCATGGACTGTGGTCATGA-3’	60.40

### Statistical analyses

GraphPad Prism 8 was used for the statistical analysis of data and figure production. All data were assessed for normality using a Shapiro-Wilk test. One-way ANOVA test was used to compare the data collected in probe trials, Open Field Test (OFT), Elevated Plus Maze (EPM), and gene expression, and pairwise comparisons between the groups were done using Tukey’s post hoc test. Values are expressed as mean ± standard deviation (SD), with *P* < 0.05 considered statistically significant.

## Results

### Open field test

**Fig. 2:**
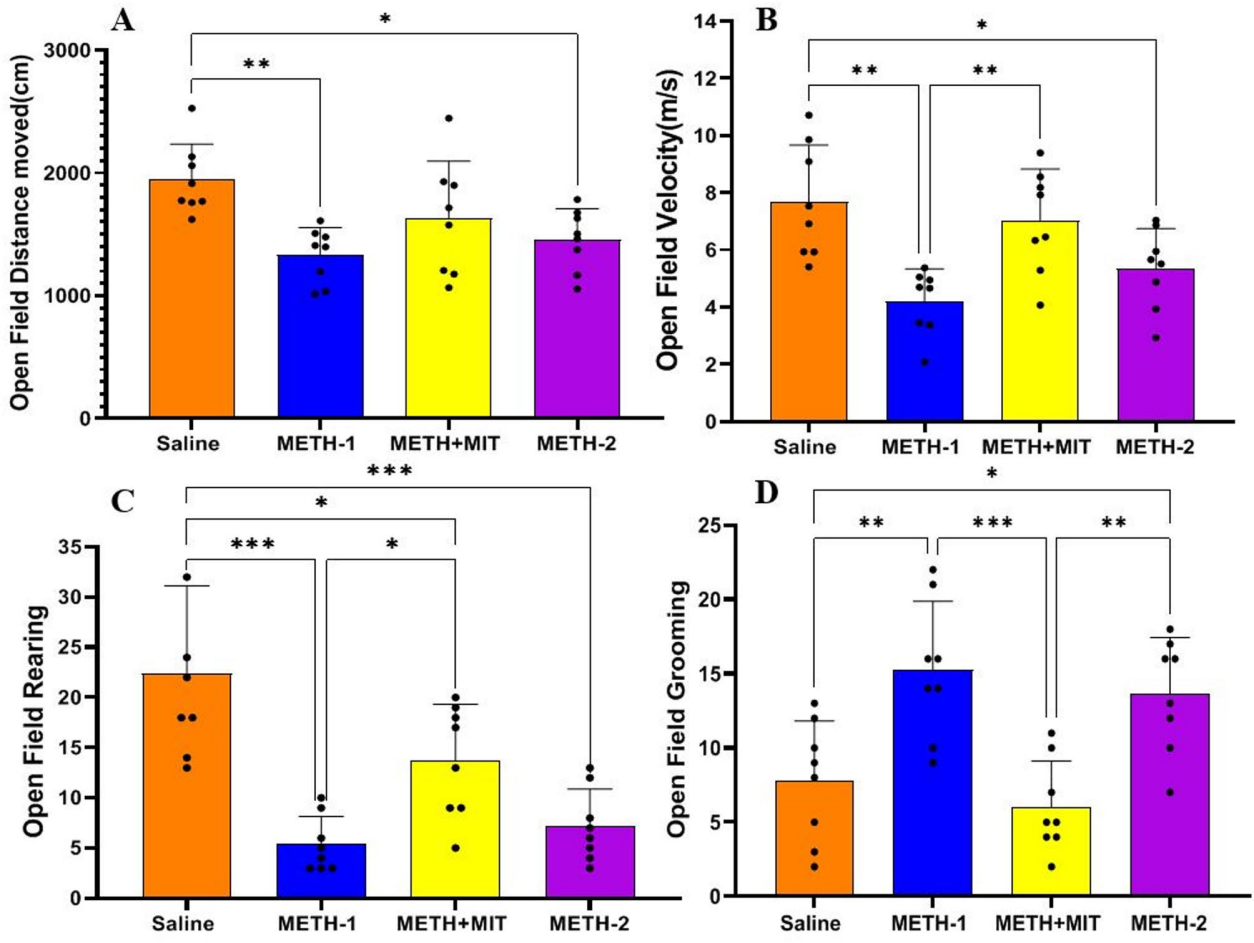
Open field test parameters in rats treated with methamphetamine (*n* = 8 per group). (A) total distance traveled, (B) movement speed, (C) rearing, (D) grooming. Statistical significance is denoted as follows: *p < 0.05; **p < 0.01; ***p < 0.001. Data are shown as mean ± standard deviation in each group; one-way ANOVA followed by Tukey’s test.

The statistical data of the OFT are presented in Fig. 2. Significant differences were observed among the groups in total distance traveled (F_3,28_ = 5.42, *P* = 0.005), movement speed (F_3,28_ = 7.67, *P* = 0.001), rearing (F_3,28_ = 14.71, *P* < 0.001), and grooming behavior (F_3,28_ = 10.31, *P* < 0.001), as a result of the interventions applied.

METH administration significantly reduced total distance traveled (METH-1 vs. saline, 95% CI = 173.2–1054, *P* = 0.004), decreased movement speed (METH-1 vs. saline, 95% CI = 1.266–5.674, *P* = 0.001), lowered rearing frequency (METH-1 vs. saline, 95% CI = 9.266–24.73, *P* = 0.001), and increased grooming behavior (METH-1 vs. saline, 95% CI = − 12.88 to − 2.120, *P* = 0.004). In contrast, exercise training exerted a beneficial effect by significantly reducing grooming behavior in the METH + MIT group compared with METH-2 (95% CI = − 13.01 to − 2.245, *P* = 0.003).

### Elevated plus maze test

**Fig. 3:**
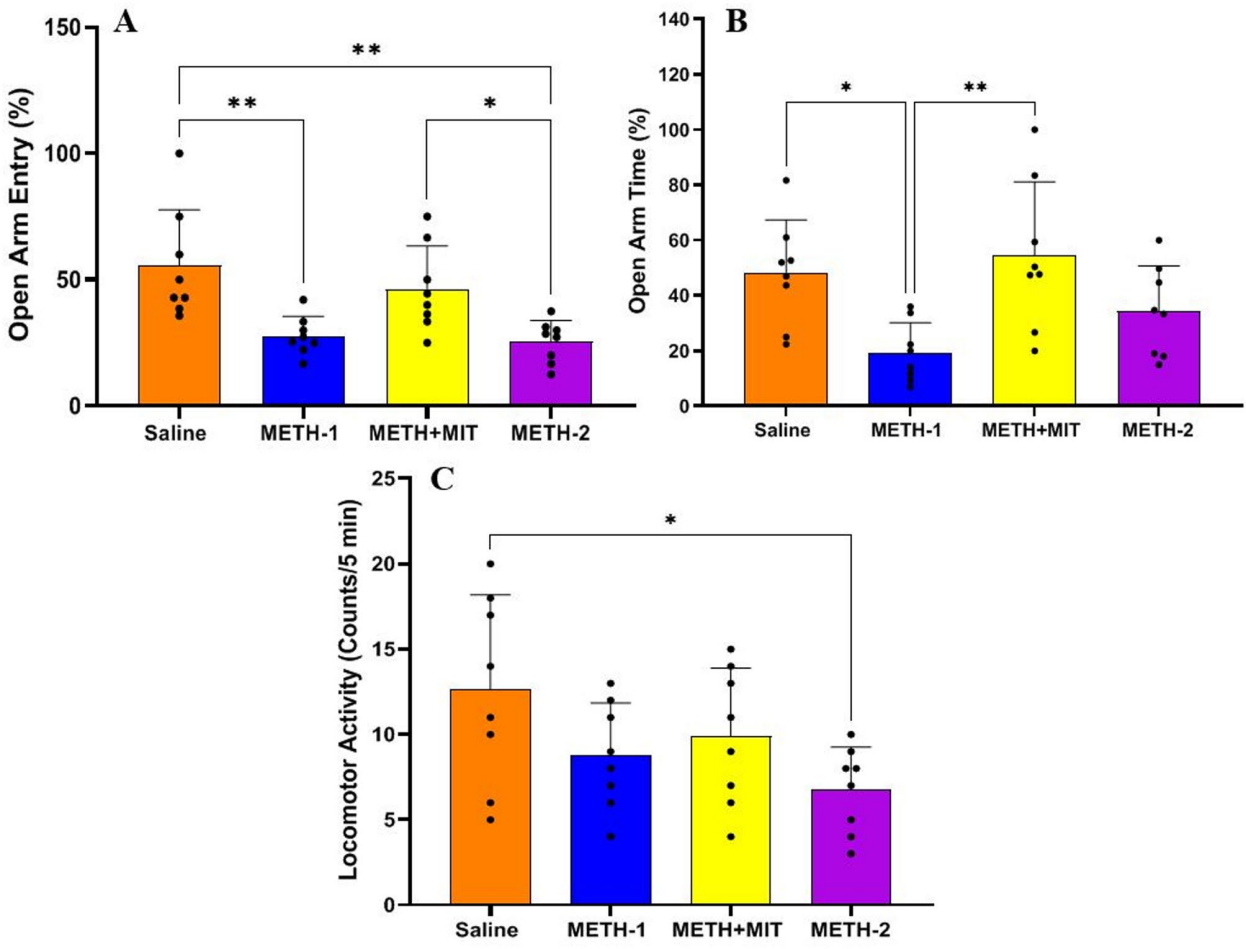
Parameters of elevated plus maze test in rats treated with methamphetamine (*n* = 8 per group). (**A**) the percentage of open arm entries, (**B**) the percentage of time spent in the open arms, (**C**) locomotor activity. Statistical significance is denoted as follows: *p < 0.05; **p < 0.01; ***p < 0.001. Data are shown as mean ± standard deviation in each group; one-way ANOVA followed by Tukey’s test.

The statistical data of the EPM are presented in Fig. 3. Significant differences were observed among the groups in the percentage of open arm entries (F_3,28_ = 7.553, *P* < 0.001), the percentage of time spent in the open arms (F_3,28_ = 5.328, *P* = 0.005), and locomotor activity (F_3,28_ = 3.062, *P* = 0.044), as a result of the applied interventions.

METH administration significantly reduced the percentage of open arm entries in the METH-1 group compared with the saline group (95% CI = 7.341–48.36, *P* = 0.005) and also significantly decreased the percentage of time spent in the open arms in the METH-1 group compared with saline (95% CI = 2.790–55.06, *P* = 0.026). In contrast, exercise training exerted a beneficial effect by significantly increasing the percentage of open arm entries in the METH + MIT group compared with the METH-2 group (95% CI = 0.3787–41.40, *P* = 0.045).

### Gene expression

**Fig. 4:**
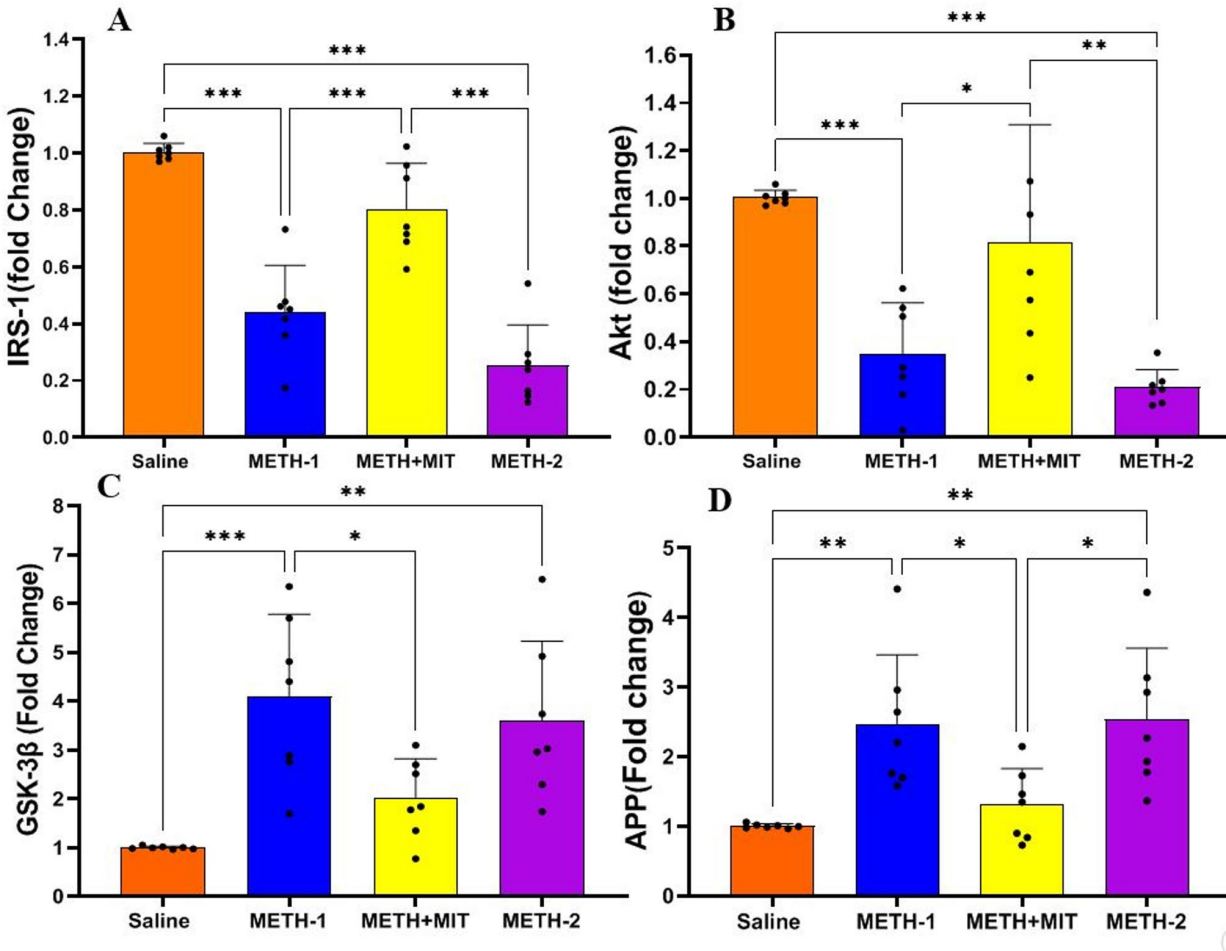
Gene expression levels of IRS‑1 (**A**), Akt (**B**), GSK‑3β (**C**), and APP (**D**) in rats treated with methamphetamine (*n* = 7 per group). Statistical significance is denoted as follows: *p < 0.05; **p < 0.01; ***p < 0.001. Data are shown as mean ± standard deviation in each group; one-way ANOVA followed by Tukey’s test.

**Fig. 5:**
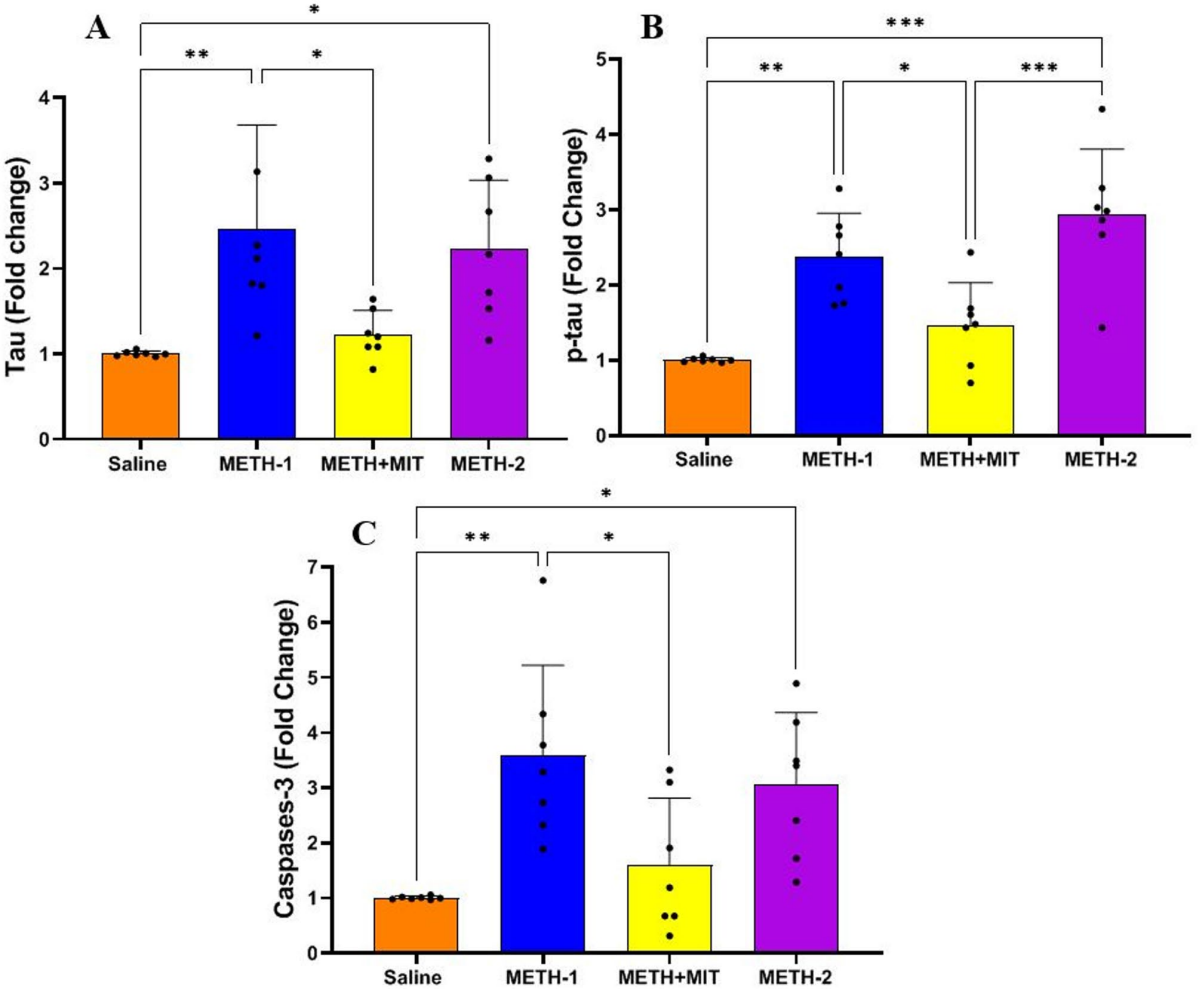
Gene expression levels of tau (**A**), p‑tau (**B**), and caspase‑3 (**C**) in rats treated with methamphetamine (*n* = 7 per group). Statistical significance is denoted as follows: *p < 0.05; **p < 0.01; ***p < 0.001. Data are shown as mean ± standard deviation in each group; one-way ANOVA followed by Tukey’s test.

The statistical data of gene expression are presented in Figs. 4 and 5, where significant differences were observed among the groups in the expression of IRS-1 (F_3,24_ = 43.93, *P* < 0.001), Akt (F_3,24_ = 13.29, *P* < 0.001), GSK-3β (F_3,24_ = 9.155, *P* < 0.001), APP (F_3,24_ = 7.535, *P* = 0.001), Tau (F_3,24_ = 6.62, *P* = 0.002), p-tau (F_3,24_ = 15.35, *P* < 0.001), and Caspase-3 (F_3,24_ = 7.01, *P* = 0.002) genes, as a result of the applied interventions.

METH administration significantly reduced the IRS-1 gene expression in the METH-1 group compared with saline (95% CI = 0.3645–0.7660, *P* < 0.001) and also significantly decreased the Akt gene expression (95% CI = 0.2553–1.061, *P* < 0.001). In contrast, METH administration significantly increased the expression of GSK-3β (95% CI = − 4.922 to − 1.249, *P* < 0.001), APP (95% CI = − 2.583 to − 0.3431, *P* = 0.007), Tau (95% CI = − 2.556 to − 0.3651, *P* = 0.006), p-tau (95% CI = − 2.236 to − 0.4959, *P* < 0.001), and Caspase-3 (95% CI = − 4.366 to − 0.7980, *P* = 0.003) genes in the METH-1 group compared with saline. Exercise training exerted beneficial effects by significantly increasing the IRS-1 gene expression in the METH + MIT group compared with METH-2 (95% CI = 0.3503–0.7515, *P* < 0.001) and by significantly increasing the Akt gene expression (95% CI = 0.2006–1.006, *P* = 0.002). Exercise also significantly reduced the expression of APP (95% CI = − 2.351 to − 0.1106, *P* = 0.028) and p-tau (95% CI = − 2.345 to − 0.605, *P* < 0.001) genes in the METH + MIT group compared with METH-2.

### Histopathological examination

**Fig. 6:**
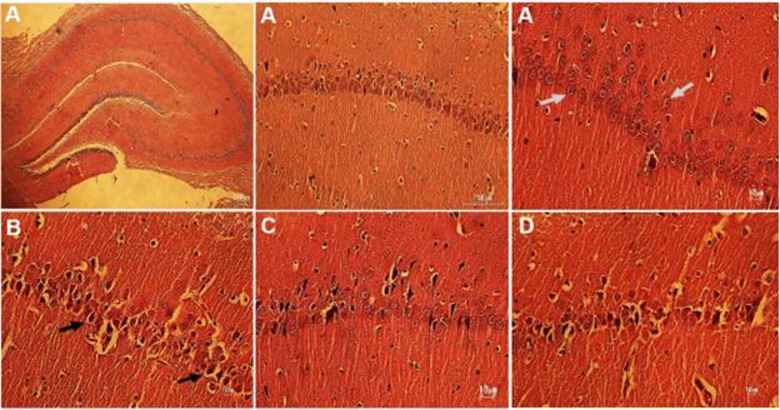
Microscopic image of hippocampal CA1 region of saline (**A**), METH-1 (**B**), METH + MIT (**C**), and METH-2 (**D**). The histological structure of the CA1 area of the hippocampus is shown the white arrow shows the healthy neuron and the black arrow shows the degenerated neuron. H&E: (40x).

**Fig. 7:**
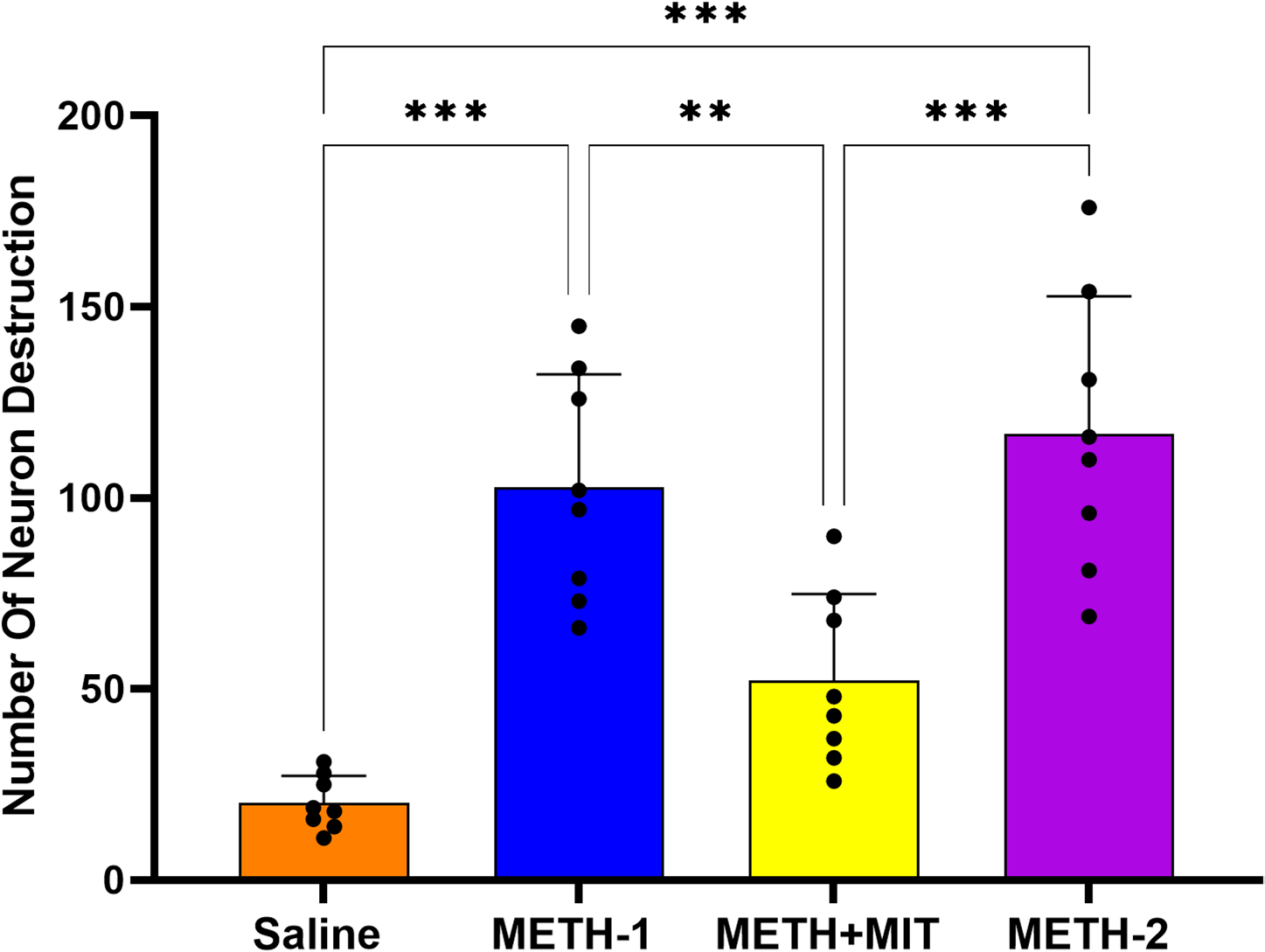
Histopathological examination of CA1 region of the hippocampus of rats treated with methamphetamine (*n* = 8 per group). Statistical significance is denoted as follows: *p < 0.05; **p < 0.01; ***p < 0.001. Data are shown as mean ± standard deviation in each group; one-way ANOVA followed by Tukey’s test.

Microscopic image of the hippocampal CA1 region of the studied groups is presented in Fig. 6. The statistical data of neuronal degeneration are presented in Fig. 7. Significant differences were observed among the groups in the histopathological assessment of neuronal degeneration in the hippocampal CA1 region (F_3,28_ = 23.34, *P* < 0.001), as a result of the applied interventions.

METH administration significantly increased neuronal degeneration in the METH-1 group compared with saline (95% CI = − 118.2 to − 46.75, *P* < 0.001). In contrast, exercise training significantly reduced neuronal degeneration in the METH + MIT group compared with the METH-2 (95% CI = − 100.1 to − 28.63, *P* < 0.001).

## Discussion

This study demonstrated that METH administration impairs locomotor activity, increases anxiety-like behavior, and induces molecular changes associated with neurodegeneration in rats. Behavioral testing using the open field and EPM paradigm significant impairments in motor activity, exploratory behavior, and anxiety-related responses following METH exposure. Specifically, reductions in locomotor activity and rearing behavior, along with increased anxiety-like behavior, were observed in METH-1 group. Importantly, MIT in METH-treated rats led to an improvement in number of self-grooming and frequency of open arm entries. These results suggest that MIT may counteract METH-induced neurobehavioral impairments.

At the molecular level, METH exposure significantly decreased hippocampal expression of insulin signaling pathway components IRS-1 and Akt, while increasing the expression of GSK-3β, APP, tau, p-tau, and Caspase-3 genes. These changes are indicative of insulin signaling disruption and neurodegeneration. Notably, MIT reversed many of these molecular alterations, restoring IRS-1 and Akt expression and reducing the expression of APP and p-tau genes, suggesting a neuroprotective role for exercise.

Previous studies have established a link between METH exposure and neurodegenerative processes such as Alzheimer’s and Parkinson’s in humans^17^. In rats, long-term METH use has been shown to promote memory loss, confusion, and dopaminergic and serotonergic neurotoxicity^18^. Additionally, METH has been found to reduce spatial and non-spatial memory due to hippocampal atrophy and neuron apoptosis^19^. METH injection disrupts the insulin signaling pathway by increasing the expression of GSK-3β, ultimately leading to an increase in APP and p-tau, which is consistent with a study by Chen et al.^3^. In AD, neuron loss occurs in several key areas of learning and memory, particularly in the hippocampus, where the PI3K/Akt pathway plays an important role in protecting neurons and neurocognitive functions related to learning and memory^20^. METH-induced inhibition of this pathway, particularly through suppression of Akt, likely contributes to neurodegeneration and behavioral decline^21^. Our study confirms this disruption and highlights that MIT may exert its benefits by reactivating the IRS-1/PI3K/Akt pathway. Our findings suggest that physical activity may be a potential intervention to prevent the accumulation of APP. This is consistent with evidence that physical activity can reduce Aβ accumulation in the brain by modulating APP metabolism and increasing the destruction and clearance of Aβ^8^. In this context, aerobic training decreased Aβ and tau protein concentrations in Alzheimer’s model mice^9^. Probably, the modulatory influence of exercise on APP metabolism is mediated by the IRS‑1/PI3K/Akt signaling cascade. Following middle cerebral artery occlusion in rats, aerobic exercise induced a significant decrease in acetylated tau protein and phosphorylated GSK3βY216, concurrently promoting an increase in IRS-1 protein, thereby highlighting its modulatory role in neuroprotection^10^.

Recent reports have shown that repeated exposure of rats to METH can lead to anxiolytic effect. Re et al.^22^ reported that two weeks of METH treatment reduced the total distance traveled in the OFT and lowered both the percentage of open arm residence time and open arm entries in the EPM. Thanos et al.^23^ reported that 16 weeks of METH administration disrupted open field activity, impaired exploratory alertness, and induced anxiety-like behavior in rats, accompanied by reduced dopamine transporter levels in the striatum. Instead, in rats subjected to METH exposure, moderate-intensity treadmill training appears to alleviate anxiety-like responses^22^.

METH may have caused oxidative stress in the hippocampus, thereby initiating intrinsic and extrinsic apoptotic pathways that culminated in neuronal loss^24^. Zhao et al.^25^ demonstrated that METH administration elevated expression of cleaved-caspase‑3 and Poly (ADP-ribose) polymerase (PARP) protein, increased apoptotic neurons in the hippocampus, and was associated with higher malondialdehyde (MDA) levels and reduced superoxide dismutase (SOD) activity. Aerobic exercise led to a marked decrease in caspase‑3 gene expression and MDA levels, along with an increase in Bcl‑2 levels in methamphetamine-dependent rats^12,24^. Histopathological studies in the CA1 region of the hippocampus of rats also showed that METH injection led to the destruction of neurons, which could be prevented to some extent by MIT.

A limitation of this study is that memory and learning in rats were not tested using the Y-Maze or Morris water maze.

Our findings indicate that METH exposure disrupts hippocampal insulin signaling, as evidenced by elevated levels of GSK-3β, APP, tau, and p-tau. Conversely, our results demonstrate that MIT enhances insulin signaling and alleviates anxiety-like behaviors. Therefore, MIT may potentially be used in humans with METH addiction to prevent neuronal destruction and improve anxiety-like behaviors, thereby reducing its adverse effects on hippocampal tissue. However, the implementation of exercise as a therapeutic strategy in substance use disorders requires further investigation. Future research should aim to define optimal exercise parameters, including intensity, frequency, duration, and modality, tailored to individuals with varying degrees and types of addiction. Moreover, well-designed clinical trials in human populations are essential to validate these preclinical findings and to develop evidence-based exercise guidelines for neuroprotection and cognitive rehabilitation in people affected by stimulant use disorders.

## Data Availability

The data is available under reasonable request to the corresponding author.

## References

[1] LaBossier, N. J. & Hadland, S. E. Stimulant misuse among youth. *Curr. Probl. Pediatr. Adolesc. Health Care*. **52**, 101265 (2022). 36184490 10.1016/j.cppeds.2022.101265 PMC10102888

[2] Rashidi, S. K. et al. Methamphetamine and the brain: Emerging molecular targets and signaling pathways involved in neurotoxicity. *Toxin Reviews*. **43**, 553–571 (2024).

[3] Chen, L. et al. Methamphetamine exposure upregulates the amyloid precursor protein and hyperphosphorylated tau expression: The roles of insulin signaling in SH-SY5Y cell line. *J. Toxicol. Sci.* **44**, 493–503 (2019). 31270305 10.2131/jts.44.493

[4] Shpakov, A. O., Zorina, I. I. & Derkach, K. V. Hot Spots for the Use of Intranasal Insulin: Cerebral Ischemia, Brain Injury, Diabetes Mellitus, Endocrine Disorders and Postoperative Delirium. *Int. J. Mol. Sci.* **24**, 3278 (2023). 36834685 10.3390/ijms24043278 PMC9962062

[5] Su, W., Wang, Y., Shao, S. & Ye, X. Crocin ameliorates neuroinflammation and cognitive impairment in mice with Alzheimer’s disease by activating PI3K/AKT pathway. *Brain Behav.* **14**, e3503 (2024). 38775292 10.1002/brb3.3503 PMC11110482

[6] Orobets, K. S. & Karamyshev, A. L. Amyloid Precursor Protein and Alzheimer’s Disease. *Int. J. Mol. Sci.* **24**, 14794 (2023). 37834241 10.3390/ijms241914794 PMC10573485

[7] Rebolledo-Pérez, L. et al. Substance Abuse and Cognitive Decline: The Critical Role of Tau Protein as a Potential Biomarker. *Int. J. Mol. Sci.* **26**, 7638 (2025). 40806766 10.3390/ijms26157638 PMC12347763

[8] Kang, J. et al. Exercise training exerts beneficial effects on Alzheimer’s disease through multiple signaling pathways. *Front. Aging Neurosci.* **17**, 1558078 (2025). 40469843 10.3389/fnagi.2025.1558078 PMC12133837

[9] Xu, L. et al. Treadmill exercise promotes E3 ubiquitin ligase to remove amyloid β and P-tau and improve cognitive ability in APP/PS1 transgenic mice. *J. Neuroinflammation*. **19**, 243 (2022). 36195875 10.1186/s12974-022-02607-7 PMC9531430

[10] Mankhong, S. et al. Effects of Aerobic Exercise on Tau and Related Proteins in Rats with the Middle Cerebral Artery Occlusion. *Int. J. Mol. Sci.* **21**, 5842 (2020). 32823945 10.3390/ijms21165842 PMC7461507

[11] Shafiei, A. et al. Effects of Moderate-Intensity Interval Training on Gene Expression and Antioxidant Status in the Hippocampus of Methamphetamine-Dependent Rats. *Neurotox. Res.* **40**, 1455–1463 (2022). 35781220 10.1007/s12640-022-00532-4

[12] Shahrabadi, H. et al. Effect of High-Intensity Interval Training on Cardiac Apoptosis Markers in Methamphetamine-Dependent Rats. *Curr. Issues Mol. Biol.* **44**, 3030–3038 (2022). 35877433 10.3390/cimb44070209 PMC9315973

[13] Li, H. Z. et al. Luteolin Enhances Choroid Plexus 5-MTHF Brain Transport to Promote Hippocampal Neurogenesis in LOD Rats. *Front. Pharmacol.* **13**, 826568 (2022). 35401160 10.3389/fphar.2022.826568 PMC8993213

[14] Pires, G. N., Tufik, S. & Andersen, M. L. Effects of sleep restriction during pregnancy on postpartum maternal behavior in female rats. *Behav. Processes*. **179**, 104200 (2020). 32710991 10.1016/j.beproc.2020.104200

[15] Song, X. et al. Transcutaneous auricular vagus nerve stimulation alleviates inflammation-induced depression by modulating peripheral-central inflammatory cytokines and the NF-κB pathway in rats. *Front. Immunol.* **16**, 1536056 (2025). 40453075 10.3389/fimmu.2025.1536056 PMC12122300

[16] Ru, Q. et al. Krill Oil Alleviated Methamphetamine-Induced Memory Impairment via the MAPK Signaling Pathway and Dopaminergic Synapse Pathway. *Front. Pharmacol.* **12**, 756822 (2021). 34776973 10.3389/fphar.2021.756822 PMC8586701

[17] Shukla, M. & Vincent, B. The multi-faceted impact of methamphetamine on Alzheimer’s disease: From a triggering role to a possible therapeutic use. *Ageing Res. Rev.* **60**, 101062 (2020). 32304732 10.1016/j.arr.2020.101062

[18] Anyanwu, G. E. et al. Morin Mitigates Methamphetamine-Induced Neurotoxicity: Effects on Motor and Cognitive Function. *J. Exp. Pharmacol.* **17**, 307–321 (2025). 40524867 10.2147/JEP.S498984 PMC12168913

[19] Shafahi, M., Vaezi, G., Shajiee, H., Sharafi, S. & Khaksari, M. Effects of crocin on learning, spatial memory impairment and necrosis cells death in rats hippocampus area in methamphetamine induced neurotoxicity. *J. Knowl. Health* **14**, 12–21 (2019).

[20] Hayati, M., Zarghoshi, J., Dabirifar, G., Yousefi, M. & Omidi, M. The effect of different training periods on beta-amyloid 42 index in hippocampus of streptozotocin-induced diabetic male rats. *Sport Physiol. Manage. Investigations*. **13**, 127–137 (2021).

[21] Omidvari, S. et al. Molecular mechanisms and treatment strategies for methamphetamine–induced neurodegeneration, inflammation and neurotoxicity. *Acta Neurobiol. Exp. (Wars)*. **83**, 414–431 (2023). 38224280 10.55782/ane-2023-2488

[22] Re, G. F. et al. Exercise modulates central and peripheral inflammatory responses and ameliorates methamphetamine-induced anxiety-like symptoms in mice. *Front. Mol. Neurosci.* **15**, 955799 (2022). 36106141 10.3389/fnmol.2022.955799 PMC9465459

[23] Thanos, P. K. et al. Effects of chronic methamphetamine on psychomotor and cognitive functions and dopamine signaling in the brain. *Behav. Brain Res.* **320**, 282–290 (2017). 27993694 10.1016/j.bbr.2016.12.010

[24] Ababzadeh, S. et al. Exercise-mediated modulation of hippocampal apoptotic gene expression and behavioral outcomes in methamphetamine-dependent rats. *Naunyn Schmiedebergs Arch. Pharmacol.* **399**, 603–612 (2025). 10.1007/s00210-025-04450-y 40643651

[25] Zhao, X. et al. Salidroside Alleviates Methamphetamine-Induced Cognitive Impairment by Targeting the AKT Pathway to Reduce Neuronal Apoptosis and Neuroinflammation. *Neuropharmacol. Therapy*. **2**, 43–58 (2025).

